# The evolving landscape of platelet therapy: risks, innovations, and clinical judgment

**DOI:** 10.1007/s00277-026-06758-y

**Published:** 2026-01-15

**Authors:** Ali Mushtaq, Moises Salgado de la Mora, Al-Homam Dabaliz, Zaher Otrock, Deborah Tolich, Moises Auron

**Affiliations:** 1 Department of Internal Medicine, Cleveland Clinic, Cleveland, OH USA; 2 Harvard T.H. Chan School of Public Health, Harvard University, Boston, MA USA; 3 College of Medicine, Alfaisal University, Riyadh, Saudi Arabia; 4 Department of Pathology and Laboratory Medicine, Diagnostics Institute Cleveland Clinic, Cleveland, OH USA; 5 Cleveland Clinic Lerner College of Medicine of Case Western Reserve University, Cleveland, OH USA

**Keywords:** Platelet transfusion, Transfusion medicine, Transfusion thresholds, Thrombocytopenia, Antiplatelet agents

## Abstract

Platelet transfusion is a cornerstone of modern supportive care, yet its application is characterized by significant practice variation and uncertainty regarding optimal strategies. This comprehensive review synthesizes current evidence to delineate a more nuanced, physiologically informed approach to platelet therapy. A paradigm shift is underway, moving from uniform count-based triggers toward more restrictive, evidence-based practices; this includes prophylactic thresholds of < 10 × 10⁹/L in stable hematology-oncology patients and therapeutic-only strategies in select populations. In massive hemorrhage, fixed-ratio resuscitation protocols incorporating early platelet administration are critical for improving hemostasis. Conversely, high-quality evidence now defines populations where transfusion may be harmful, including in thrombotic microangiopathies like TTP, heparin-induced thrombocytopenia, and spontaneous intracerebral hemorrhage in patients on antiplatelet agents. The utility of viscoelastic assays in guiding goal-directed therapy and the potential of novel products such as pathogen-reduced, cold-stored, and cryopreserved platelets to mitigate the limitations of conventional storage are also critically examined. This review provides clinicians with a framework to navigate these complexities, emphasizing a context-dependent strategy that balances hemostatic benefit against potential harm to optimize patient outcomes and steward a precious resource.

## Introduction

Platelets are essential cellular components of blood, playing a critical role in hemostasis and preventing hemorrhage following vascular injury or dysfunction. Platelets are a lifesaving but exceptionally precious resource, and with a shelf-life of only five to seven days, maintaining an adequate and safe supply is a constant logistical challenge for blood services worldwide [1]. However, they serve as a crucial intervention option for patients with thrombocytopenia and platelet dysfunction to mitigate bleeding risk. The use of platelet transfusions has increased significantly over the past two decades, underscoring their importance in various clinical settings [2, 3]. Despite their established role, considerable Uncertainty remains about the optimal use of platelet transfusions and the most appropriate transfusion thresholds across various clinical scenarios. While the Association for the Advancement of Blood & Biotherapies (AABB) recommends prophylactic platelet transfusions for non-bleeding patients with hypoproliferative thrombocytopenia and a platelet count below 10 × 10^9^/L, guidance for many other clinical scenarios is either lacking or supported by weak recommendations, leading to significant variability in transfusion practices [4].

Emerging evidence from recent clinical trials has revealed significant controversies and uncertainties regarding the risk-benefit profile of platelet transfusions in various patient populations [5, 6]. These findings underscore the need to expand our understanding of platelet function beyond their role in hemostasis, recognizing their involvement in inflammation and immune modulation [7]. To optimize the value of platelet transfusions, opportunities exist in developing more restrictive transfusion policies and exploring alternative strategies for their use. These include innovations such as cold-stored and cryopreserved platelets, which may offer enhanced hemostatic function and a longer shelf life compared to conventional platelets [8, 9]. 

This article serves as a comprehensive narrative review that aims to serve as a detailed, evidence-based resource for clinicians navigating the complexities of platelet transfusion therapy in their daily practice. In addition to examining current indications and emerging alternatives, we will address critical topics such as platelet transfusion refractoriness, the influence of product type and dosing, the use of platelets in special populations, including pregnancy and cirrhosis, and contraindications for platelet transfusion in select clinical situations.

## Pathophysiology of thrombocytopenia and bleeding risk

**Fig. 1:**
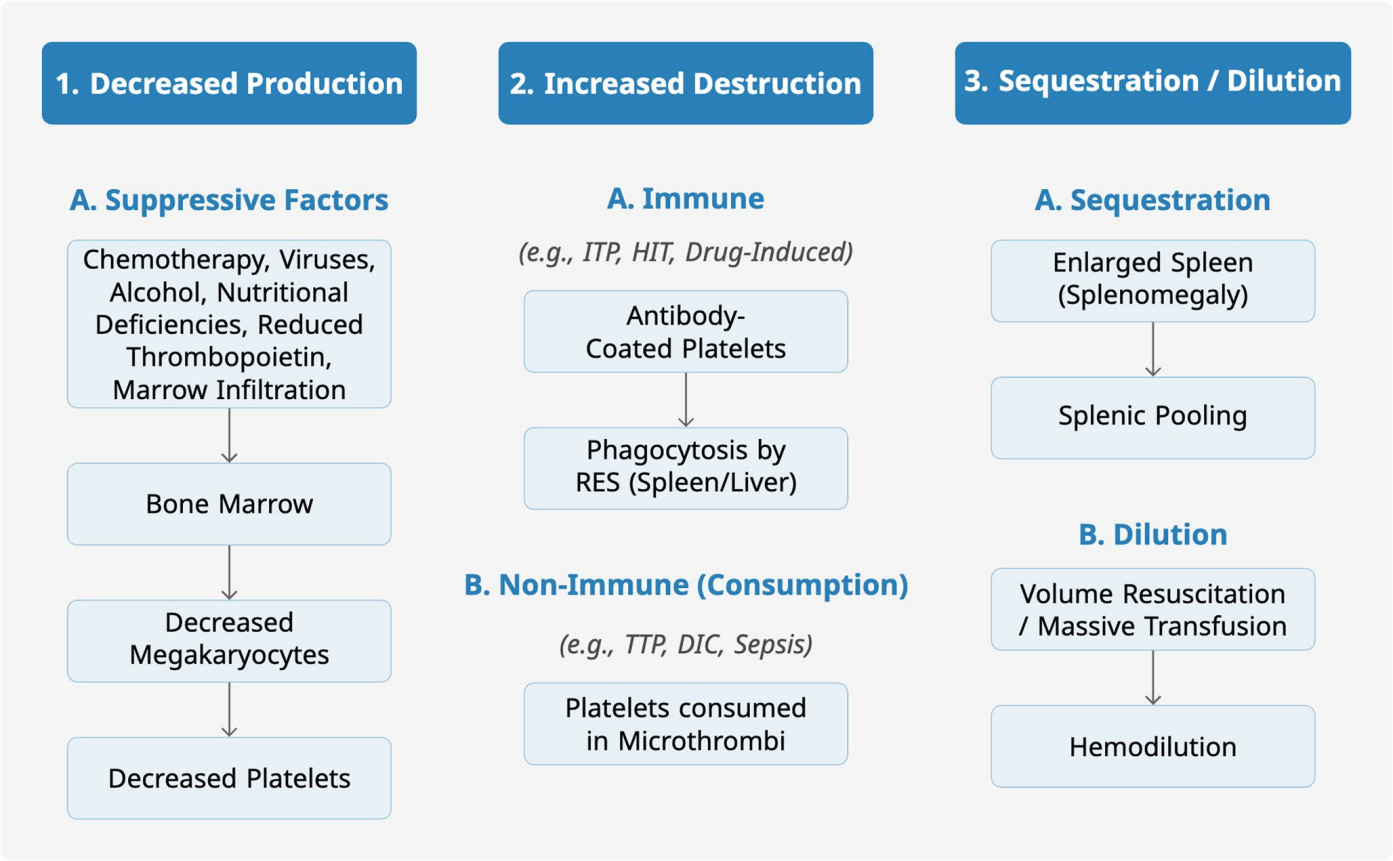
Primary Mechanisms for Thrombocytopenia

Platelets are derived from megakaryocytes, which originate from multipotent hematopoietic stem cells in the bone marrow [10]. Thrombopoietin is the key growth factor driving megakaryocyte development and platelet production [11, 12]. A single mature megakaryocyte can release 2,000 to 5,000 platelets, [13] resulting in the generation of approximately 10¹¹ new platelets per day [14]. An approach to platelet transfusion necessitates a fundamental understanding of the etiologies of thrombocytopenia and its clinical consequences. Thrombocytopenia, defined as a subnormal platelet count (most often as < 150,000/µL), represents a common endpoint of diverse pathological processes. The risk of hemorrhage, however, is not solely determined by platelet count; it involves a complex interplay of platelet number and function, vascular endothelial integrity, and the coagulation cascade [15, 16]. While the prevailing clinical assumption posits a direct link between thrombocytopenia and bleeding, and that platelet transfusion mitigates this risk, the underlying cause of thrombocytopenia profoundly influences the actual bleeding risk. For example, most individuals would remain asymptomatic as long as their platelet count exceeds 50,000/µL [17]. However, patients with counts below 30,000/µL may experience bleeding from minimal trauma, and those with counts below 10,000/µL may experience spontaneous bleeding, especially in mucosal surfaces [18]. The three primary mechanisms for thrombocytopenia are decreased bone marrow production, sequestration, and increased platelet destruction [17]. See Fig. 1 for the pathophysiology of thrombocytopenia and how to characterize it.

### Decreased platelet production

Many malignancies, such as leukemias, lymphomas, and solid cancers (prostate and breast), as well as myeloid disorders, including myeloproliferative syndromes and myelodysplastic syndromes, may lead to the replacement of the bone marrow with either malignant or fibrotic infiltrations, resulting in decreased production of all blood cell lines [17]. Thrombocytopenia secondary to bone marrow suppression also occurs as a consequence of viral infections. Alternatively, many viruses, such as the human immunodeficiency virus (HIV) and hepatitis C, have been associated with thrombocytopenia due to bone marrow suppression, as well as certain medications, chemotherapy, and alcohol [19, 20].

### Increased platelet destruction or consumption

Beyond insufficient production, accelerated platelet destruction or consumption represents a significant cause of thrombocytopenia. This process can be immune-mediated, as observed in immune thrombocytopenia (ITP) and heparin-induced thrombocytopenia (HIT) [21], where antibodies facilitate premature platelet clearance by the reticuloendothelial system [22, 23]. Alternatively, non-immune consumption characterizes conditions like disseminated intravascular coagulation (DIC), sepsis, and thrombotic microangiopathies such as thrombotic thrombocytopenic purpura (TTP), [24, 25] where widespread coagulation activation and endothelial damage lead to rapid platelet consumption, resulting in the formation of microthrombi. The often newly released and hyper-reactive nature of platelets in these consumptive states further complicates the straightforward interpretation of platelet counts as indicators of bleeding risk. Other conditions, such as systemic lupus erythematosus and sarcoidosis, can also cause thrombocytopenia through immune-mediated destruction [26, 27].

### Sequestration and dilution

Thrombocytopenia in patients with portal hypertension as well as malignancies often stems from splenic sequestration, where a significant portion of the platelet pool is retained within an enlarged spleen, leading to lower peripheral counts despite adequate marrow production. Concurrently, dilutional thrombocytopenia is a well-recognized phenomenon in the context of massive bleeding and resuscitation, as the infusion of platelet-poor fluids, such as packed red blood cells (RBCs) and crystalloids, rapidly lowers the concentration of circulating platelets [28, 29]. These sequestered platelets may also reduce platelet production in the bone marrow by removing thrombopoietin from the circulation and lowering its levels [30]. 

The occurrence of thrombocytopenia is notable during pregnancy, representing the second most common hematological derangement [17]. It is crucial to differentiate between the benign gestational thrombocytopenia, which is a physiological consequence of plasma volume expansion [18], and thrombocytopenia associated with more severe pregnancy-related conditions. Specifically, a depressed platelet count is a common feature in both preeclampsia and eclampsia, and a profound decrease in platelets is a defining criterion for HELLP syndrome (Hemolysis, Elevated Liver enzymes, and Low Platelets) [31].

### Drug-induced thrombocytopenia

Drug-induced thrombocytopenia manifests through varied pathophysiological pathways, primarily classified as immune-mediated or non-immune-mediated. The immune-mediated form involves the development of drug-dependent antibodies that selectively bind to platelet glycoproteins when the inciting drug is present, resulting in the rapid clearance of platelets from the circulation. Prominent examples include thrombocytopenia induced by trimethoprim-sulfamethoxazole, penicillin, carbamazepine, ceftriaxone, ibuprofen, mirtazapine, and the glycoprotein IIb/IIIa inhibitors abciximab, tirofiban, and eptifibatide [32]. A critical variant, heparin-induced thrombocytopenia (HIT), involves antibodies directed against the platelet factor 4 (PF4)-heparin complex, uniquely resulting in profound platelet activation and an elevated risk of thrombosis, rather than bleeding [20, 33]. In contrast to immune mechanisms, nonimmune drug-induced thrombocytopenia (DIT) results from the direct toxicity of certain drugs on platelets, frequently in a dose-dependent manner. Agents such as mitomycin C, alpha-interferon, tacrolimus, and various chemotherapeutics and immunosuppressants are implicated [34]. Clinically, this form of DIT can closely mimic TTP [35].

### Chemotherapy-Induced Thrombocytopenia (CIT)

Chemotherapy-induced thrombocytopenia (CIT) is a frequent and dose-limiting toxicity of many cytotoxic regimens, particularly those containing platinum agents, gemcitabine, and temozolomide. The resulting thrombocytopenia not only increases the patient’s risk of bleeding but often forces dose reductions or delays in treatment, which may compromise oncologic outcomes [36, 37]. The traditional management for severe CIT or active bleeding relies on prophylactic platelet transfusions, but these offer only a transient solution.

To provide more durable platelet support and maintain chemotherapy schedules, thrombopoietin receptor agonists (TPO-RAs) have been investigated. Recent meta-analyses and trials have demonstrated their efficacy; eltrombopag is effective in reducing chemotherapy dose modifications, recombinant human thrombopoietin (rhTPO) reduces the incidence of severe (grade 3/4) thrombocytopenia, and romiplostim has shown high response rates (up to 85%) in correcting platelet counts to allow for chemotherapy resumption [37–40]. The primary limitation of these agents is an increased risk of venous thromboembolism, a significant concern in patients with malignancies who already have an elevated baseline thrombotic risk (Tables 1 and 2).

**Table 1:** Platelet transfusion-associated adverse reactions: types, incidence, and mitigation strategies

Adverse Reaction	Rate per platelet transfused*	Common Signs and Symptoms	Mitigation Strategies	Reference
Allergic	10–30/1000	Pruritus; flushing; conjunctival edema; edema of lips, tongue and uvula; periorbital erythema and edema; maculopapular rash; urticaria (hives); bronchospasm; localized angioedema	Slow transfusion rate (mild reactions), antihistamines, plasma-reduced products	(1) (2) (3) (4)
Anaphylactic	0.02–0.05/1000	Hypotension, shock, angioedema, wheezing, stridor, dyspnea, respiratory distress, abdominal pain, vomiting, loss of consciousness	Intramuscular epinephrine, antihistamines, glucocorticoids, bronchodilators, vasopressors, plasma-reduced products, IgA-deficient products (patients with IgA deficiency), autologous donation for planned procedures	(1) (3) (4)
Febrile nonhemolytic	1–10/1000	Fever (≥ 38 deg C) or chills/rigors	Antipyretics, leukocyte-reduced blood products	(1) (4) (5)
TAD	NA	Dyspnea, tachypnea, hypoxemia	Respiratory support	(1) (6)
Hypotensive transfusion reaction	NA	Hypotension (within an hour of transfusion); other possible symptoms include facial flushing, dyspnea, or abdominal cramps	Stop transfusion (typically resolves within 10 min), Trendelenburg position	(1) (2) (3)
TRALI	0.03/1000	Acute respiratory failure, hypoxemia, dyspnea, hypotension, fever, bilateral pulmonary infiltrates	Cardiovascular and respiratory support; diuretics may help	(1) (6) (7) (8)
Acute hemolytic transfusion reaction (AHTR)	NA	Fever, chills, flank/back pain, hypotension, hemoglobinuria, disseminated intravascular coagulation, acute renal failure, shock	Cardiovascular, respiratory, and renal support, hemostatic blood components for bleeding	(1) (3) (9)
Septic	≤ 0.1/1000	Fever, chills, rigors, hypotension	Stop the transfusion, antibiotics	(1)
TACO	2.6/1000	Dyspnea, orthopnea, cough, headache, tachycardia, hypertension, pulmonary edema, positive fluid balance	Upright posture, oxygen, diuretics, and a slow transfusion rate	(1) (6) (7) (8)
Posttransfusion Purpura (PTP)	NA	Purpura, severe thrombocytopenia, bleeding	Intravenous immunoglobulin (IVIG), plasma exchange, and glucocorticoids	(1) (3)
Transfusion-Associated Graft-versus-Host Disease (TA-GVHD)	NA	Maculopapular rash, fever, watery diarrhea, nausea, vomiting, abdominal pain, hepatomegaly, liver dysfunction, pancytopenia	Irradiation of cellular blood products for patients at risk (e.g., immunocompromised)	(1) (3)

Abbreviations: *NA*, not available; *TACO*, Transfusion-Associated Circulatory Overload; *TAD*, Transfusion-Associated Dyspnea; *TRALI*, Transfusion-Related Acute Lung Injury

*Data from the 2025 AABB and ICTMG International Clinical Practice Guidelines

1. Abdallah R, Rai H, Panch SR. Transfusion Reactions and Adverse Events. Clin Lab Med. 2021; 41(4):669-96.

2. Soutar R, McSporran W, Tomlinson T, Booth C, Grey S. Guideline on the investigation and management of acute transfusion reactions. Br J Haematol. 2023; 201(5):832-44.

3. Delaney M, Wendel S, Bercovitz RS, Cid J, Cohn C, Dunbar NM, et al. Transfusion reactions: prevention, diagnosis, and treatment. Lancet. 2016;388(10061):2825-36.

4. Goel R, Tobian AAR, Shaz BH. Noninfectious transfusion-associated adverse events and their mitigation strategies. Blood. 2019;133(17):1831-9.

5. Wang H, Ren D, Sun H, Liu J. Research progress on febrile non-hemolytic transfusion reaction: a narrative review. Ann Transl Med. 2022;10(24):1401.

6. Grey S, Bolton-Maggs P. Pulmonary complications of transfusion: Changes, challenges, and future directions. Transfus Med. 2020;30(6):442-9.

7. Semple JW, Rebetz J, Kapur R. Transfusion-associated circulatory overload and transfusion-related acute lung injury. Blood. 2019;133(17):1840-53.

8. van den Akker TA, Grimes ZM, Friedman MT. Transfusion-Associated Circulatory Overload and Transfusion-Related Acute Lung Injury. Am J Clin Pathol. 2021; 156(4):529-39.

9. Panch SR, Montemayor-Garcia C, Klein HG. Hemolytic Transfusion Reactions. N Engl J Med. 2019; 381(2):150-62.

**Table 2:** Summary of prophylactic platelet transfusion thresholds (adopted from reference #4)

Procedure	Platelet Threshold (x 10^9^/L)	Strength of Recommendation/Evidence Quality	Source(s)
Nonbleeding patients with hypoproliferative thrombocytopenia actively receiving chemotherapy or undergoing allogeneic stem cell transplantation	< 10	Strong/Moderate	(1)
Non-bleeding adults with hypoproliferative thrombocytopenia undergoing autologous stem cell transplantation or diagnosed with aplastic anemia	Not recommended	Conditional/Low to very low	(1)
Central venous catheter insertion at sites that may be manually compressed	< 10	Conditional/Moderate to very low	(1), (2) (3)
Lumbar puncture	< 20	Strong/Moderate	(1)
Major non-neuraxial surgery	< 50	Conditional/Very low	(1) (4)
CNS or ophthalmologic surgery	< 100	Strong/Low quality	(5)
Bone marrow aspirate or trephine biopsy	Not recommended	Strong/Moderate	(5)
Cardiothoracic surgery, including those receiving cardiopulmonary bypass (without thrombocytopenia or bleeding)	Not recommended	Conditional/Very low	(1)
Interventional radiology procedures (non-liver disease)	Low Risk < 20 High Risk < 50	Conditional/Very low	(1)

1. Metcalf RA, Nahirniak S, Guyatt G, Bathla A, White SK, Al-Riyami AZ, et al. Platelet Transfusion: 2025 AABB and ICTMG International Clinical Practice Guidelines. Jama. 2025

2. Kaufman RM, Djulbegovic B, Gernsheimer T, Kleinman S, Tinmouth AT, Capocelli KE, et al. Platelet transfusion: a clinical practice guideline from the AABB. Ann Intern Med. 2015;162(3):205 − 13

3. van Baarle FLF, van de Weerdt EK, van der Velden W, Ruiterkamp RA, Tuinman PR, Ypma PF, et al. Platelet Transfusion before CVC Placement in Patients with Thrombocytopenia. N Engl J Med. 2023; 388(21):1956-65

4. Patel IJ, Rahim S, Davidson JC, Hanks SE, Tam AL, Walker TG, et al. Society of Interventional Radiology Consensus Guidelines for the Periprocedural Management of Thrombotic and Bleeding Risk in Patients Undergoing Percutaneous Image-Guided Interventions-Part II: Recommendations: Endorsed by the Canadian Association for Interventional Radiology and the Cardiovascular and Interventional Radiological Society of Europe. J Vasc Interv Radiol. 2019;30(8):1168-84.e1

5. Estcourt LJ, Birchall J, Allard S, Bassey SJ, Hersey P, Kerr JP, et al. Guidelines for the use of platelet transfusions. Br J Haematol. 2017; 176(3):365 − 94

### Acquired platelet dysfunction

Acquired platelet dysfunction, a qualitative defect in platelet function distinct from inherited disorders, impairs platelet adhesion, aggregation, secretion, or procoagulant activity, often leading to mucocutaneous bleeding despite normal platelet counts and morphology [41]. Diagnosis typically relies on platelet function testing, with light transmission aggregometry being the most common method; automated aggregometry is also rapidly evolving [42]. While medications, particularly antiplatelet agents (e.g., aspirin, NSAIDs, P2Y12 inhibitors) and newer drugs like ibrutinib, are the most frequent causes, other etiologies include dietary factors, systemic medical disorders (e.g., uremia, liver disease, myeloproliferative neoplasms), and certain procedures (e.g., cardiopulmonary bypass, extracorporeal membrane oxygenation). Less commonly, autoimmune mechanisms, infections, or hematologic malignancies can also induce acquired platelet dysfunction [43, 44]. Table 3 provides a differential for the etiologies of thrombocytopenia.

**Table 3:** Etiologies of thrombocytopenia and acquired platelet dysfunction

Primary Mechanism	Sub-Mechanism/Category	Examples & Specific Etiologies	References
Decreased Platelet Production	Bone Marrow Suppression/Infiltration	- Viruses (e.g., HIV, hepatitis C) - Malignancies (leukemias, lymphomas, solid cancers like prostate and breast) - Myeloid disorders (myeloproliferative syndromes, myelodysplastic syndromes)	[19, 20]
Increased Platelet Destruction or Consumption	Immune-Mediated	- Immune thrombocytopenia - Heparin-induced thrombocytopenia - Systemic lupus erythematosus, sarcoidosis	[45]
	Non-Immune Consumption	- Disseminated intravascular coagulation - Sepsis - Thrombotic microangiopathies (e.g., TTP)	[46]
Sequestration and Dilution	Splenic Sequestration	- Portal hypertension with an enlarged spleen	[28]
	Dilutional	- Massive bleeding and resuscitation with platelet-poor fluids (packed red blood cells, crystalloids)	[29]
	Pregnancy-Related	- Gestational thrombocytopenia - Preeclampsia and eclampsia - HELLP syndrome	[18, 31]
Drug-Induced Thrombocytopenia (DIT)	Immune-Mediated	- Drug-dependent antibodies (e.g., trimethoprim-sulfamethoxazole, penicillin, ceftriaxone, ibuprofen) - A specific variant is HIT, with antibodies against the PF4-heparin complex	[20, 32, 33]
	Non-Immune (Direct Toxicity)	- Dose-dependent toxicity from agents like mitomycin C, alpha-interferon, tacrolimus, and various chemotherapeutics	[34]
Acquired Platelet Dysfunction	Qualitative Defect	- Medications (e.g., aspirin, NSAIDs, P2Y12 inhibitors, ibrutinib) - Systemic disorders (e.g., uremia, liver disease, myeloproliferative neoplasms) - Procedures (e.g., cardiopulmonary bypass, ECMO)	[43, 44]

Abbreviations: TTP: Thrombotic Microangiopathy; HELLP Syndrome: Hemolysis, Elevated Liver enzymes, and Low Platelet count (Syndrome); P2Y12 Inhibitors: Platelet P2Y12 Receptor Blockers; ECM: Extracorporeal Membrane Oxygenation

## Platelet preparation

Platelet products are administered either as pooled platelet concentrates or, more commonly, as apheresis platelets. A therapeutic dose for adults contains approximately 3 to 4 × 10^11^ platelets [47]. This can be achieved by pooling four to six whole blood-derived platelet concentrate units. A single apheresis procedure typically yields a higher platelet count, and it can usually be divided into two or three therapeutic doses [48]. In clinical practice, one therapeutic adult dose is expected to increase the platelet count by more than 10 × 10^9^/L. [49]. The largest and most robust clinical trial to date, the Platelet Dose (PLADO) trial, found no significant difference in bleeding risk among low-dose (1.1 × 10^11^/m^2^), standard-dose (2.2 × 10^11^/m^2^), and high-dose (4.4 × 10^11^/m^2^) platelet transfusions. However, patients in the low-dose group required significantly more transfusions than those in the standard- and high-dose groups [50]. 

**Fig. 2:**
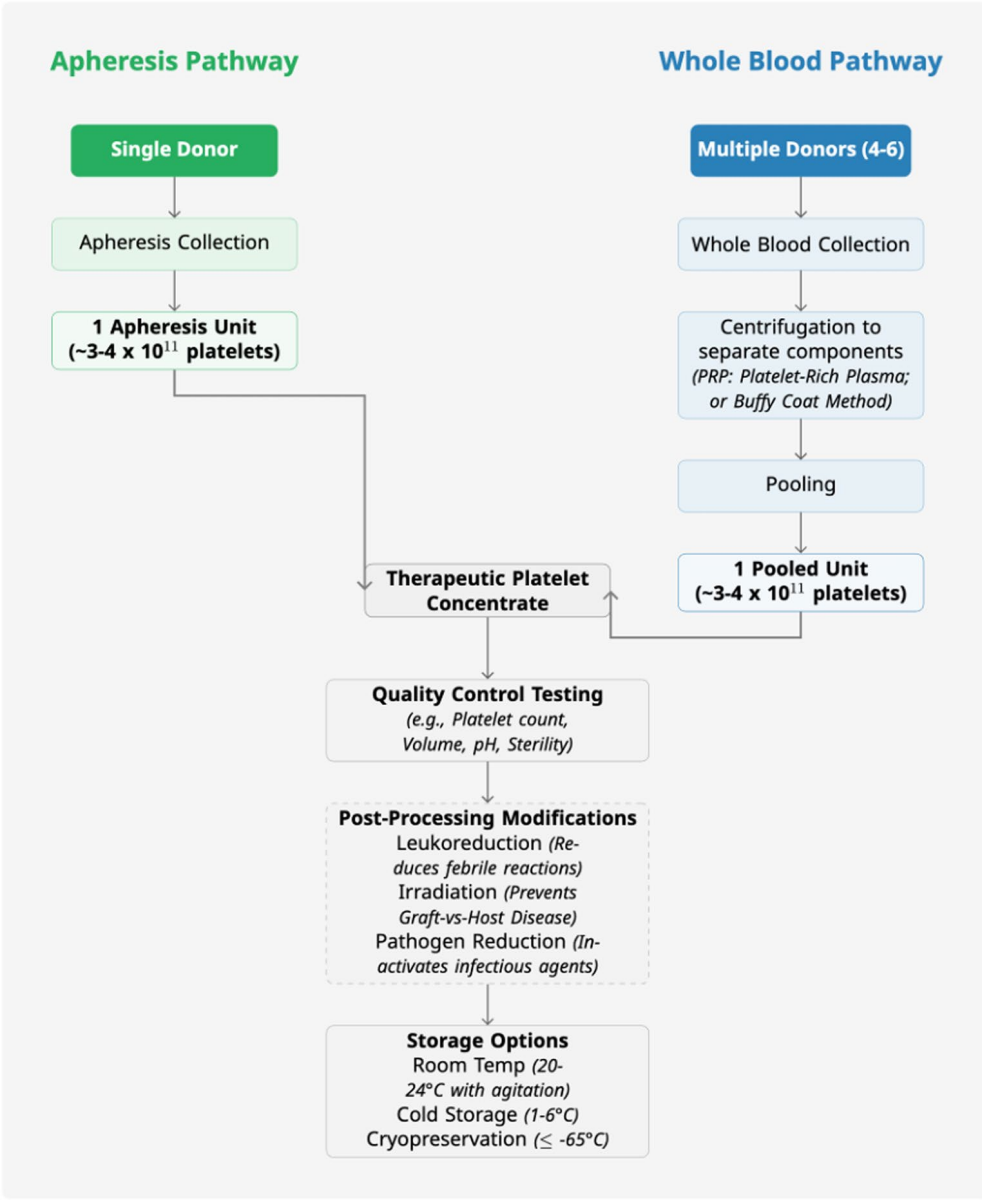
Comparison of Apheresis-Derived vs. Whole Blood-Derived Platelet Components

Despite the singular nomenclature, platelet transfusion encompasses a heterogeneous array of products. Variations stemming from collection and processing methods, the implementation of pathogen-inactivation strategies, and specific storage conditions each contribute to distinct platelet unit characteristics and associated risk-benefit profiles. Recognizing these fundamental differences is crucial for tailoring transfusion therapy to meet the precise needs of individual patients. See Fig. 2 for more details on platelet manufacturing and processing.

### Collection

There are three primary methods for collecting platelets from donors. Among them, the platelet-rich plasma (PRP) and the buffy coat (BC) methods use whole blood as the source material [51]. Both methods require pooling multiple units of blood from various donors, as a single unit contains an insufficient quantity for a therapeutic effect in adults. These are less expensive and easier to produce; however, donor matching is not possible, which increases the risk of HLA alloimmunization, especially since platelets from 4 to 6 different donors must be pooled together [52]. The third method collects platelets alone from the donor via apheresis and returns the remainder of the blood cells to the donor. In this method, a full adult patient platelet dose can be collected from a single donor [51]. Apheresis units minimize donor exposure, which is crucial for patients requiring repeated transfusions, but are more resource-intensive to collect. Many studies have been conducted to explore the clinical differences between these methods. Apheresis has been shown to allow for more prolonged survival of platelets compared to pooled platelets [53]. When it comes to safety, the nature of pooled platelets, which come from multiple donors, appears to lead to a higher incidence of viral transmission and bacterial contamination [54]. The PLADO trial, involving over 1200 patients, found no significant difference in transfusion-related adverse events or bleeding prevention efficacy between leukoreduced apheresis and whole blood-derived platelets in a post hoc analysis [55]. While a 2008 systematic review of ten randomized controlled trials noted higher corrected count increments with apheresis platelets, it concluded that definitive clinical benefit differences between these leukoreduced products remain unproven due to limitations in trial size and quality [56]. 

### Processing

After platelet collection, specific procedures are performed on the collected sample, primarily aimed at reducing the risk of transfusion reactions and complications. Leukoreduction is mainly performed on pooled blood among these crucial procedures [57]. Leukoreduction aims to reduce the number of leukocytes in collected blood units by filtering them out of the unit [58]. This has proven benefits in preventing febrile non-hemolytic transfusion reactions, reducing infections from intracellular viruses like CMV, and preventing alloimmunization in transfusion recipients [59]. Irradiation, on the other hand, is explicitly done to inactivate residual lymphocytes within the collected blood [60]. The primary purpose behind this is to prevent the development of transfusion-associated graft-versus-host disease (TA-GvHD), which occurs when viable lymphocytes in the transfused component proliferate and perform an immune attack on the recipient’s tissue [61, 62]. Another procedure commonly done is pathogen reduction. This procedure aims to ensure safety and eliminate microbial contamination by treating the product with chemicals, such as amotosalen and riboflavin, in conjunction with ultraviolet light illumination. This also helps reduce the risk of TA-GvHD by targeting lymphocytes within the product. However, this procedure does inflict some damage to the platelets, but this has not been attributed to cause any serious risks in recipients [63–65]. Platelet additive solutions (PAS) may also be used to reduce the risk of transfusion reactions. In PAS, the plasma is replaced with a solution that can improve platelet storage. These solutions contain various ingredients, including acetate, magnesium, potassium, and sodium. Studies have shown that some PAS formulations have equal or even better platelet survival compared to platelets stored in plasma. It is also noted that many transfusion reactions, like transfusion-related acute lung injury, occur less frequently in PAS [66–68]. 

### Storage

In contrast to RBCs, platelet function is compromised by storage at low temperatures. Thus, the standard storage condition for platelets is at room temperature between 20 and 24 °C with gentle shaking [1]. This allows platelets to be stored for 5 days in a closed system, with the potential to extend storage up to 7 days [69]. However, this practice inadvertently creates an environment highly conducive to bacterial proliferation and associated septic complications. In contrast, cold storage (1–6 °C) is emerging as a compelling alternative, offering a significant reduction in the risk of bacterial contamination, as discussed later in this manuscript [70]. 

## Platelet transfusion-associated adverse events

Although beneficial primarily to patients, platelet transfusions are associated with the highest rate of adverse reactions [71]. The most commonly recorded reactions to platelets are allergic reactions and febrile non-hemolytic transfusion reactions. Less common reactions have also been observed after platelet transfusion, such as hypertensive and hypotensive reactions, transfusion-associated circulatory overload (TACO), transfusion-associated dyspnea (TAD), and transfusion-related acute lung injury (TRALI) [72–74]. 

This underscores that platelet transfusions, though valuable, carry a notable risk of complications. Table 1 provides a detailed overview of platelet transfusion-associated adverse reactions.

## Current evidence for prophylactic platelet transfusion

The practice of prophylactic platelet transfusion, which involves administering platelets to a non-bleeding, thrombocytopenic patient to prevent hemorrhage, has been a central focus of transfusion medicine research for decades. The goal is to maintain a platelet count above a theoretical hemostatic threshold, thereby reducing the risk of spontaneous bleeding. This principle has been most rigorously studied in patients with bone marrow transplant and hematological malignancies [75–77]. However, its application has extended to nearly every facet of clinical medicine, and evidence remains inconclusive for other thrombocytopenic populations, presenting a clinical dilemma.

The AABB offers updated specific recommendations for prophylactic platelet transfusions. These include transfusions at platelet counts < 10 × 10⁹/L for nonbleeding patients with hypoproliferative thrombocytopenia actively receiving chemotherapy or undergoing allogeneic stem cell transplant, < 10 × 10⁹/L for central venous catheter placement at anatomic sites amenable to manual compression, < 20 × 10⁹/L for lumbar punctures, major non-neuraxial surgeries < 50 × 10^9^/L, and no transfusions for non-thrombocytopenic patients undergoing cardiac surgery [4]. Prophylactic platelet transfusions have been studied in thrombocytopenic patients undergoing invasive procedures. A recent randomized controlled trial demonstrated a reduction in bleeding with the use of prophylactic platelet transfusions during ultrasound-guided central venous catheter placement [78]. Similarly, the International Society of Interventional Radiology provides guidance on platelet thresholds for various percutaneous image-guided procedures [79, 80]. Table 2 provides a summary of prophylactic platelet transfusion thresholds for various clinical scenarios.

Leading the shift in platelet transfusion strategies, the TOPPS trial [81] and a notable German study by Wandt et al.[82] consistently demonstrated the safety and efficacy of a “no-prophylaxis” or therapeutic-only approach compared to a standard prophylactic transfusion strategy (at a 10 × 10³/µL trigger) in patients with thrombocytopenia.

They reported more frequent minor bleeding events (WHO Grade 2) in the no-prophylaxis arms; critically, there was no significant increase in major or life-threatening hemorrhage (WHO Grade 3 or 4) [77, 82]. In the TOPPS trial, 50% of patients in the no-prophylaxis group experienced WHO grade 2, 3, or 4 bleeding, compared to 43% in the prophylaxis group (*P* = 0.06 for non-inferiority), with no significant difference in major bleeding rates. The Wandt study similarly found increased grade 2 or greater bleeding in the therapeutic-only group (42% vs. 19% in the prophylactic group, *P* < 0.001), but significant (grade 3/4) bleeding was rare and not significantly different in autologous transplant recipients [81, 82]. 

This acceptance of minor bleeding risk translated to approximately 33–50% fewer platelet transfusions for patients in the no-prophylaxis groups, a pivotal finding that informed the conditional recommendation in the 2025 guidelines for a no-prophylaxis strategy in adult patients with aplastic anemia or those undergoing autologous stem cell transplant, thus representing a significant advancement toward high-value, patient-centered care [4]. 

Prophylactic platelet transfusions in critically ill patients often yield suboptimal responses. For example, a study reported a poor incremental platelet response in 73.9% of cases, with maximum storage duration independently associated with poor outcomes [83]. The response was particularly poor in patients with hematological malignancies receiving chemotherapy, occurring in 93.5% of cases. The PLOT-ICU study, a multicenter investigation across the U.S. and Europe, assessed platelet transfusion practices in ICU patients with thrombocytopenia (platelet count < 150 × 10⁹/L). The study found that approximately 21% of patients received transfusions, with prophylactic transfusions demonstrating limited increases in platelet count. Additionally, wide variations in dosing highlighted the need for standardization and further research [84]. 

The PlaNet-2/MATISSE trial explored platelet transfusion thresholds in preterm neonates, revealing surprising results. A restrictive threshold of < 25 × 10⁹/L was associated with significant reductions in major bleeding and mortality compared to a threshold of < 50 × 10⁹/L, resulting in a 7% absolute risk reduction [85]. These findings suggest that a lower threshold of < 25 × 10⁹/L may benefit all preterm neonates, regardless of predicted bleeding or mortality risk [86, 87]. 

Evidence continues to evolve regarding optimal thresholds for prophylactic platelet transfusions in various patient populations. Current research highlights the need for better standardization of transfusion practices and further investigation into subgroup-specific risks and benefits. Additionally, emerging data suggest that restrictive thresholds may be advantageous in specific populations, such as preterm neonates, underscoring the importance of tailoring transfusion strategies to individual patient needs.

## Current evidence for therapeutic platelet transfusion in active bleeding

Despite considerable, albeit incomplete, evidence guiding prophylactic transfusions, the more urgent scenario of active bleeding presents a paradoxical lack of high-quality data to inform transfusion decisions. While the clinical goal of achieving immediate hemostasis is clear, the ethical and logistical challenges of conducting randomized controlled trials in actively bleeding patients are challenging. Consequently, current practice is predominantly shaped by physiological principles, institutional guidelines, and expert opinion.

### Massive hemorrhage and trauma

In the setting of massive hemorrhage, frequently defined in the context of trauma, the paramount objective is to prevent or reverse the lethal triad of acidosis, hypothermia, and coagulopathy. This imperative has driven the development of Massive Transfusion Protocols, advocating for balanced, empiric resuscitation with blood components. The concept of fixed-ratio transfusion, which aims to reconstitute whole blood composition with ratios such as 1:1:1 (plasma: platelets: RBCs), has gained significant traction. This approach was supported by the landmark PROPPR (Pragmatic, Randomized Optimal Platelet and Plasma Ratios) trial. This multicenter, phase 3 randomized controlled trial compared 1:1:1 and 1:1:2 ratios in 680 severely traumatized patients predicted to require massive transfusion. While PROPPR did not demonstrate a significant difference in 24-hour all-cause mortality, patients receiving the 1:1:1 ratio achieved hemostasis more rapidly and exhibited a significant reduction in death from exsanguination within the first 24 h. These findings strongly endorse the early use of higher amounts of platelets as an integral part of a balanced resuscitation strategy [88]. 

These findings were further supported by a meta-analysis showing that higher platelet-to-RBC ratios are associated with significantly lower short-term and 30-day mortality in massively transfused trauma patients [89]. Therefore, the recommendation is to target a high ratio of plasma and platelets to RBCs for resuscitating severely injured bleeding trauma patients, ideally in a 1:1:1 ratio to optimize hemostasis and improve survival outcomes [90].

### General therapeutic transfusions

The severity and site of bleeding guide the current evidence for therapeutic platelet transfusion in active bleeding [91]. For stable, non-bleeding patients with therapy-induced hypoproliferative thrombocytopenia, a platelet transfusion threshold of < 10 × 10^3^/µL is typically appropriate [4]. This threshold increases to < 50 × 10^3^/µL for actively bleeding patients. For high-risk anatomical sites, such as the central nervous system or neurosurgical procedures, a more stringent threshold of < 100 × 10^3^/µL is recommended. Invasive procedures warrant transfusion at 20 − 50 × 10^3^/µL, depending on the associated bleeding risk. In conditions like DIC or other high-risk bleeding scenarios, a range of 30 − 50 × 10^3^/µL is commonly employed [92–94]. 

Platelet transfusion in patients with intracerebral hemorrhage (ICH) on antiplatelet therapy remains controversial. The PATCH trial showed that transfusing platelets in such patients—despite normal platelet counts—led to worse functional outcomes and increased death or dependence at 3 months [95]. A supporting meta-analysis by Lin et al. confirmed that platelet transfusions do not improve outcomes or reduce mortality in this setting [6]. As a result, the 2025 AABB/ICTMG guidelines conditionally recommend against platelet transfusion in non-operative ICH patients with platelet counts > 100 × 10³/µL, even if they are on antiplatelet therapy [4]. These findings underscore that transfusing platelets in an inflammatory environment may cause more harm than benefit.

### Special considerations

#### Disseminated intravascular coagulation

Disseminated intravascular Coagulation (DIC) represents a profound challenge in critical care, characterized by a paradoxical and life-threatening duality: diffuse microvascular thrombosis coupled with severe hemorrhagic diathesis. This consumptive coagulopathy, driven by an uncontrolled activation of coagulation, depletes platelets and clotting factors, leading to widespread organ dysfunction and a high mortality rate. Current management hinges on the aggressive identification and definitive treatment of the underlying etiology (e.g., sepsis, trauma, malignancy), as supportive transfusion therapy alone is insufficient to halt disease progression. Consequently, the optimal role of platelet transfusion remains highly contentious and would only be indicated in patients with active bleeding, or to maintain a threshold of 20–30 × 10⁹/L may be considered [46, 96]. 

#### Prothrombotic and immune-mediated thrombocytopenia

In thrombotic thrombocytopenic purpura (TTP), a deficiency in the ADAMTS13 enzyme leads to the spontaneous formation of platelet-rich microthrombi throughout the microvasculature. This widespread clotting consumes platelets, leading to thrombocytopenia. Consequently, platelet transfusion is strictly contraindicated in TTP, unless there is truly life-threatening hemorrhage. Providing exogenous platelets in this context can exacerbate organ damage by supplying more substrate for these pathological clots. Instead, the cornerstone of TTP management is plasma exchange and potent immunosuppression, which aim to restore ADAMTS13 activity and halt the thrombotic process [97]. 

Similarly, heparin-induced thrombocytopenia (HIT) is a profoundly prothrombotic disorder, despite the falling platelet count. It is driven by antibodies that aberrantly activate platelets, leading to an elevated risk of both arterial and venous thrombosis. Therefore, platelet transfusion is also generally contraindicated in HIT, as it can paradoxically intensify this hypercoagulable state. The essential and immediate management steps are the complete cessation of all heparin products and the prompt initiation of a non-heparin anticoagulant to mitigate the risk of thrombosis [97, 98]. 

Immune thrombocytopenia (ITP), while also immune-mediated, presents a distinct challenge. Patients with ITP often have a lower bleeding risk at a given platelet count compared to those with bone marrow failure. This is because their circulating platelets are typically younger, larger, and more hemostatically active. As such, platelet transfusions in ITP are reserved for cases of severe, life-threatening bleeding. The effect of transfused platelets is often transient, as they are rapidly cleared by the same autoantibodies responsible for the underlying thrombocytopenia. The primary goal of transfusion in ITP is not to achieve a specific numerical platelet target, but rather to provide a temporary hemostatic benefit while definitive immune-modulating therapies, such as corticosteroids or intravenous immunoglobulin, take effect [45]. 

#### Inherited platelet disorders

Glanzmann thrombasthenia (GT) and Bernard-Soulier syndrome (BSS) are rare inherited platelet function disorders that can cause bleeding despite normal platelet counts. Routine prophylactic transfusion is not required; instead, management focuses on preventing complications before high-risk procedures and treating active bleeding episodes [99]. Patients with GT and BSS have an increased risk of alloimmunization, and antibodies against the specific deficient glycoproteins can also develop. Therefore, platelet transfusion is reserved for major surgery, childbirth, or severe bleeding episodes [100]. For minor mucocutaneous bleeding, antifibrinolytics and local measures are typically effective [100]. When transfusion is necessary, leukocyte-depleted, HLA-matched platelet transfusions are preferred. Alternatively, recombinant activated factor VII (rFVIIa) is approved for patients with GT who are refractory to platelet transfusion due to the presence of antibodies, and it has also been successfully used in patients with BSS. Patients receiving platelet transfusions should be evaluated for HLA and platelet-specific antibodies 2–3 months after their last transfusion [99, 100]. 

#### Liver dysfunction

In patients with liver disease, thrombocytopenia and platelet dysfunction are also multifactorial, and bleeding episodes correlate better with platelet count than with standard coagulation measures [101]. Management of active bleeding or risk reduction before high-risk procedures involves transfusions of cryoprecipitate, platelets, and RBCs, targeting levels of fibrinogen ≥ 120 mg/dL, platelet count ≥ 50 × 10^9^/L, and hematocrit ≥ 25%, respectively [102]. However, this data has been extrapolated from the trauma literature and expert consensus, rather than from robust clinical trials.

#### Thrombocytopenia requiring concurrent anticoagulation

Managing therapeutic anticoagulation in thrombocytopenic patients presents a significant clinical challenge, especially for those with hematologic malignancies, chronic liver disease, or HIT who also have concurrent atrial fibrillation or venous thromboembolism. This situation requires carefully balancing the patient’s bleeding and thrombotic risks. There is a lack of prospective randomized data to define optimal transfusion and anticoagulation strategies in this population. Therefore, management must be individualized, weighing the VTE recurrence risk against the bleeding risk and considering patient-specific factors such as the anticipated duration and severity of thrombocytopenia [103, 104].

## Platelet transfusion refractoriness

Platelet transfusion refractoriness (PTR) is a common and complex issue in which patients fail to achieve adequate increases in platelet count after transfusion. It is often diagnosed by a corrected count increment (CCI) of less than 5 × 10⁹/L or a percentage of platelet recovery of less than 30% at one hour post-transfusion on two occasions [105, 106]. This complicates care for patients requiring ongoing platelet support, thereby heightening the risk of bleeding. PTR stems from either non-immune causes, such as fever, sepsis, or active bleeding, or immune causes, primarily alloantibodies against HLA or human platelet antigens (HPAs) that develop after prior sensitization through pregnancy, transfusions, or transplantation [107]. Differentiating between these is crucial for management, which involves addressing underlying non-immune factors or providing HLA/HPA-matched or crossmatch-compatible platelets for immune-mediated cases, which can yield adequate increments in 50–60% of transfusions [105, 108, 109]. Future therapeutic approaches for immune PTR aim to decrease platelet HLA antigen expression or mitigate antibody-mediated effects [110]. 

## Alternatives to platelet transfusion

Several alternative agents may be considered to prevent bleeding in patients who are either refractory to platelet transfusions or decline blood products (e.g., Jehovah’s Witnesses). Thrombopoietin receptor agonists, which stimulate endogenous platelet production, represent one option; however, their delayed onset of action (typically 1–2 weeks) limits their utility in acute settings [111]. Antifibrinolytic agents, such as tranexamic acid and epsilon aminocaproic acid, have demonstrated efficacy in managing active bleeding and trauma; however, evidence for their prophylactic use in thrombocytopenia remains limited [112, 113]. Other proposed therapies, including desmopressin, which is useful in inherited platelet function disorders or uremic bleeding, recombinant factor VIIa and XIII used off-label for refractory bleeding, fibrinogen concentrate, cryoprecipitate, and artificial platelet substitutes, may be considered for bleeding prevention or treatment; however, evidence remains insufficient to support their routine use [111]. 

## Novel platelet products

### Pathogen-reduced platelets

Pathogen-reduced platelets (PR platelets) are produced using technologies such as amotosalen/UVA (INTERCEPT), riboflavin/UV (Mirasol), and UVC-based systems (Theraflex), which inactivate a broad spectrum of pathogens and leukocytes [114, 115]. This significantly reduces the risk of transfusion-transmitted infections, particularly bacterial sepsis, and eliminates the risk of transfusion-associated graft-versus-host disease, while also increasing the shelf life from 5 to 7 days, a duration that some countries have adopted [115–117]. In terms of efficacy, randomized controlled trials and meta-analyses indicate that PR platelets are non-inferior to conventional platelets for preventing clinically significant or severe bleeding [116, 118]. 

One limitation is reduced post-transfusion platelet count increments noted by multiple studies [119, 120]. Furthermore, while not increasing severe adverse events, some PR technologies like the Mirasol-treated platelets are associated with a slightly higher risk of clinically significant bleeding (WHO grade ≥ 2). In contrast, INTERCEPT-treated platelets do not show a statistically significant increase in bleeding risk compared to standard platelets. However, the risk of severe bleeding (WHO grade ≥ 3) and all-cause mortality does not appear to be increased with either technology [116, 119]. They can also alter platelet metabolic function, creating a crucial clinical trade-off between enhanced safety and potentially diminished efficacy [121, 122]. 

### Cold-stored platelets

Cold-stored platelets (CSP) were the standard of care from the 1950 s until room temperature-stored platelets (RTP) were introduced in the 1970s. CSP have significantly shorter circulatory survival after transfusion—typically 1 to 3 days (half-life of 1–2 days)—compared to RTP, which last 7 to 9 days (half-life of 5–7 days) [123, 124]. The transition from CSP to RTP was primarily motivated by the need to extend posttransfusion platelet survival in patients with chronic thrombocytopenia, particularly those with hematologic and oncologic conditions [125]. 

CSP, maintained at 1–6 °C, have re-emerged as a promising alternative to RTP for the management of acute bleeding, particularly in surgical and trauma settings [126, 127]. CSP demonstrate superior or comparable hemostatic efficacy to RTP, with enhanced thrombin generation, preserved contractile function, and the ability to form occlusive thrombi under arterial shear even after extended storage (up to 14–21 days), which RTP does not match beyond 5–7 days. These properties are attributed to cold-induced metabolic and structural changes that “prime” platelets for hemostatic activity, including preserved mitochondrial integrity and reduced apoptosis [128, 129]. Additionally, refrigeration reduces the risk of bacterial contamination, extending shelf life and improving inventory management [127, 130]. However, CSP are associated with reduced post-transfusion platelet recovery and survival compared to RTP, making them less suitable for prophylactic use in chronic thrombocytopenia but potentially advantageous for acute bleeding where immediate hemostatic function is prioritized [131]. While current studies show CSP are well-tolerated with no new safety concerns, large randomized trials are needed to confirm their efficacy and safety across different patient populations [132]. 

### Cryopreserved platelets

Frozen or cryopreserved platelets are platelet concentrates preserved at −80 °C, typically using 5–6% dimethyl sulfoxide (DMSO) as a cryoprotectant, which enables storage for up to two years and addresses the logistical challenges of short shelf-life and bacterial contamination associated with RTP [133]. The development of cryopreserved platelets began over 60 years ago, with Valeri’s method establishing a reproducible protocol that remains the basis for current practice [134]. Cryopreserved platelets are particularly valuable in settings with limited access to fresh platelets, such as rural hospitals, military deployments, and during mass casualty events, due to their extended shelf life and rapid availability [133, 135]. 

Cryopreservation induces significant changes in platelet morphology and function, including reduced aggregation response to agonists and increased procoagulant activity, due to enhanced microparticle release and earlier thrombin generation [136, 137]. These properties may be advantageous in acute bleeding scenarios, such as trauma or cardiac surgery, where rapid hemostasis is critical. A recent pilot randomized controlled trial in New Zealand has demonstrated the safety of cryopreserved platelets in cardiac surgery. Beyond acute hemorrhage, their utility has been explored in chronically thrombocytopenic patients. A key prospective, randomized, crossover study in patients with hematologic malignancies found that cryopreserved platelets provided effective hemostasis and acceptable post-transfusion increments, supporting their potential use as an alternative to standard platelets, especially during inventory shortages [138]. However, post-transfusion platelet recovery is lower compared to that of liquid-stored platelets, likely due to altered glycoprotein expression and accelerated clearance from the circulation [139]. Ongoing and future randomized clinical trials are necessary to establish the clinical efficacy and safety of cryopreserved platelets in actively bleeding patients and to define their role relative to conventional platelet products.

### Lyophilized platelets

The manufacturing process preserves key surface glycoproteins and procoagulant activity, allowing reconstituted platelets to support primary hemostasis by enhancing adhesion and providing a surface for thrombin generation.

Lyophilized platelets are room-temperature-stable, freeze-dried platelet products designed to address the limitations of conventional platelet storage. The lyophilization process involves stabilizing platelets, followed by freezing and sublimation of water, resulting in a powder that can be stored at room temperature for years and rapidly reconstituted when needed. This process preserves key surface glycoproteins and procoagulant activity, allowing reconstituted platelets to support primary hemostasis [8, 140, 141]. However, this process significantly impairs their aggregation response and reduces in vivo survival compared to fresh platelets [142–144]. Although promising as a rapidly deployable hemostatic agent in trauma, surgery, or settings with limited blood banking infrastructure, these platelets remain largely investigational, with limited clinical data to establish their safety and efficacy in humans [8, 142]. 

## Conclusion

In conclusion, the practice of platelet transfusion is shifting from a simple count-based algorithm to a nuanced, evidence-based paradigm. This evolution is characterized by restrictive prophylactic strategies, balanced resuscitation in massive hemorrhage, and the recognition of contraindications where transfusion may be futile or cause harm, such as in TTP or specific cases of ICH. This precision is increasingly supported by functional viscoelastic assays and the development of novel platelet products, including pathogen-reduced, cold-stored, and cryopreserved options, designed to overcome the limitations of conventional platelet products. Clinicians must integrate a deeper understanding of hemostasis with current evidence to ensure the judicious application of this vital resource, maximizing therapeutic benefit while minimizing risk.

## Data Availability

As this is a review article, all data discussed are derived from previously published research and are fully cited within the text and reference list. No new data were created or analyzed in this study. Data sharing is not applicable to this article.
